# Serology Identifies LIPyV as a Feline Rather than a Human Polyomavirus

**DOI:** 10.3390/v15071546

**Published:** 2023-07-13

**Authors:** Sergio Kamminga, Els van der Meijden, Patricia Pesavento, Christopher B. Buck, Mariet C. W. Feltkamp

**Affiliations:** 1 Department of Medical Microbiology, Leiden University Medical Center, 2333 ZA Leiden, The Netherlandsm.c.w.feltkamp@lumc.nl (M.C.W.F.); 2 Department of Pathology, Microbiology & Immunology, University California Davis Veterinary Medicine, 5323 Vet Med 3A, Davis, CA 95616, USA; 3 Laboratory of Cellular Oncology, Center for Cancer Research, National Cancer Institute, Bethesda, MD 20892, USA; 4 Department of Donor Medicine Research, Sanquin Research, 1066 CX Amsterdam, The Netherlands

**Keywords:** Lyon IARC polyomavirus, feline virus, human polyomavirus

## Abstract

The number of identified human polyomaviruses (HPyVs) has increased steadily over the last decade. Some of the novel HPyVs have been shown to cause disease in immunocompromised individuals. The Lyon-IARC polyomavirus (LIPyV) belonging to species *Alphapolyomavirus quardecihominis* was identified in 2017 in skin and saliva samples from healthy individuals. Since its initial discovery, LIPyV has rarely been detected in human clinical samples but has been detected in faeces from cats with diarrhoea. Serological studies show low LIPyV seroprevalence in human populations. To investigate the possibility that LIPyV is a feline rather than a human polyomavirus, we compared serum IgG responses against the VP1 major capsid protein of LIPyV and 13 other HPyVs among cats (n = 40), dogs (n = 38) and humans (n = 87) using an in-house immunoassay. Seropositivity among cats was very high (92.5%) compared to dogs (31.6%) and humans (2.3%). Furthermore, the median antibody titres against LIPyV were 100–10,000x higher in cats compared to dogs and humans. In conclusion, the high prevalence and intensity of measured seroresponses suggest LIPyV to be a feline rather than a human polyomavirus. Whether LIPyV infection induces diarrhoea or other symptoms in cats remains to be established.

## 1. Introduction

Human polyomaviruses (HPyVs) are small, double-stranded DNA viruses, that cause asymptomatic, persistent infections starting at infancy. Some HPyV species cause severe disease in immunocompromised patients. Important examples include BK polyomavirus (BKPyV) and JC polyomavirus (JCPyV), which cause kidney, bladder, and brain disease in immunosuppressed individuals, and trichodysplasia spinulosa polyomavirus (TSPyV) and Merkel cell polyomavirus (MCPyV), which cause dysplastic and neoplastic skin disease, respectively (Table 1). A number of HPyVs have not yet been associated with disease [1] (Table 1).

The seroprevalence of HPyVs is usually very high in humans, ranging from 60 to 100% among adults [18,19]. Three puzzling exceptions to this general trend are human-associated HPyV12, HPyV13 (also known as New Jersey polyomavirus), and HPyV14 (also known as Lyon-IARC polyomavirus, LIPyV), which show very low seroprevalences and have rarely been detected in human clinical samples [20]. It is interesting to note that HPyV12 was discovered in samples of human liver tissue [15]. The team that discovered HPyV12 using PCR-based methods later detected a nearly identical sequence in samples of shrews (*Sorex araneus*), suggesting that the HPyV12 sequence reflected a lab environment contaminant [21]. HPyV13 was discovered in tissue samples of a transplant patient suffering from vasculitis, myositis, and retinal blindness. Although histological confirmation of the HPyV13 discovery in the index patient strongly suggests that the virus is a bona fide human-tropic virus, NJPyV DNA has not been reported in any other samples of humans or animals [16].

The alphapolyomavirus LIPyV, which shows low seroprevalence and low IgG antibody titers in humans [18], was first described in 2017, with detection in approximately 2% of collected human skin, eyebrow, and oral gargle samples [17]. More recently, LIPyV DNA has been primarily detected in faecal samples from diarrheic cats (in 2/100 and 3/5 faecal samples, respectively) [22,23] and only rarely in human samples [23,24].

It is thought that polyomaviruses generally co-evolve with host mammal groups, such that the phylogeny of polyomaviruses tends to mirror the phylogeny of host animals [25] LIPyV-like sequences have also been found in other carnivores, including raccoons and pumas [26]. In a broad survey of the NCBI Sequence Read Archive, we detected examples of novel LIPyV-related genomes in cat samples (accession numbers: BK063195, BK063196, BK063197) but none in primate samples.

Given these observations, we hypothesise that LIPyV represents a feline rather than a human-tropic polyomavirus. To test this hypothesis, we compared serum IgG antibody responses against the immunodominant Viral Protein 1 (VP1) major capsid protein of LIPyV in cats, dogs, and humans.

## 2. Materials and Methods

### 2.1. Polyomavirus Serology

A customised Luminex multiplex immunoassay was used to assess IgG antibody responses against the major capsid protein VP1 of LIPyV and 13 other HPyVs, namely BKPyV, JCPyV, Karolinska Institute polyomavirus (KIPyV), Washington University polyomavirus, MCPyV, Human polyomavirus 6 (HPyV6), Human polyomavirus 7 (HPyV7), TSPyV, Human polyomavirus 9, Malawi polyomavirus (MWPyV), Saint Louis polyomavirus (STLPyV), HPyV12, and NJPyV (Table 1). This assay was previously described in detail [27]. Briefly, VP1 fusion proteins were expressed in *E. coli* and coupled to uniquely coloured, magnetic fluorescent beads (Bio-Rad Laboratories, Hercules, CA, USA). Biotinylated goat-α-cat IgG, rabbit-α-dog IgG, and goat-α-human IgG (H+L) (Jackson ImmunoResearch, Cambridgeshire, United Kingdom, dilution 1:1000) were used as secondary conjugate antibodies that were detected with streptavidin-R-phycoerythrin (SAPE) (1:1000). Antibody responses were measured in a Bio-Plex 200 analyser (Bio-Rad Laboratories, Hercules, CA, USA) and analysed using Bio-Plex Manager 6.1 software. Specific antibody responses were calculated by subtracting from each sample the median fluorescence intensity (MFI) values of a blank sample (no serum added) and of beads coupled to SV40 small t protein as a background measurement. An arbitrary cutoff for seropositivity was set at 1500 MFI.

### 2.2. Samples and Study Populations

Forty serum samples from cats and 40 serum samples from dogs were analysed for IgG HPyV seroresponsiveness. Cat and dog serum samples were sourced with owner consent from adult cats routinely admitted for elective procedures to the William R. Pritchard Veterinary Medical Teaching Hospital (VMTH) at the University of California, Davis. A cohort of anonymised serum samples from 87 healthy blood donors from the Netherlands [28] were included in the analyses as human controls. The donors gave written informed consent, and the study adhered to the Declaration of Helsinki principles.

### 2.3. Sequence Read Archive Database Search

Diamond 2.0.15 software [29] was used in BlastX mode to scan Sequence Read Archive records representing carnivores and primates for the presence of protein sequences resembling the helicase domain of LIPyV LT antigen, with settings—block-size 7—index-chunks 1—max-target-seqs 1—evalue 0.00000000001—outfmt 6 qseqid sseqid evalue qseq. Datasets with Blast e-values less than e-50 were subjected to de novo assembly with Megahit 1.2.9 software [30]. Contigs of interest were identified and polished using CLC Genomics Workbench 22 and MacVector 18.5.1.

### 2.4. Phylogenetic Tree

A phylogenetic tree was constructed by downloading relevant genomes from NCBI RefSeq database and extracting LT sequences. These were aligned, and a phylogenetic tree was constructed using ngphylogeny.fr [31] using default parameters. The tree was further edited using the online interactive Tree of Life program [32].

### 2.5. Statistics

Descriptive statistics were performed in Graphpad Prism (version 9.0.1. GraphPad Software Inc., San Diego, CA, USA). Comparative statistics were performed with Mann–Whitney U test.

## 3. Results

**Figure 1:**
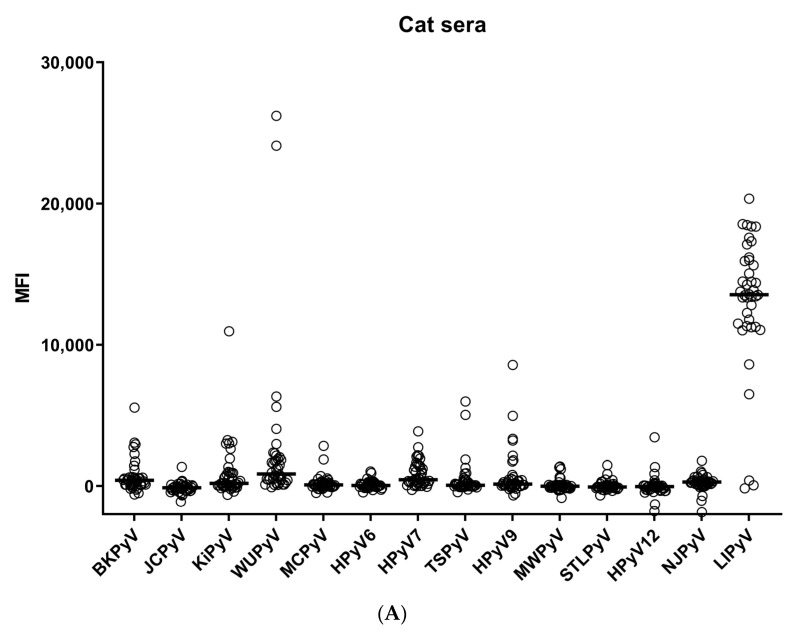

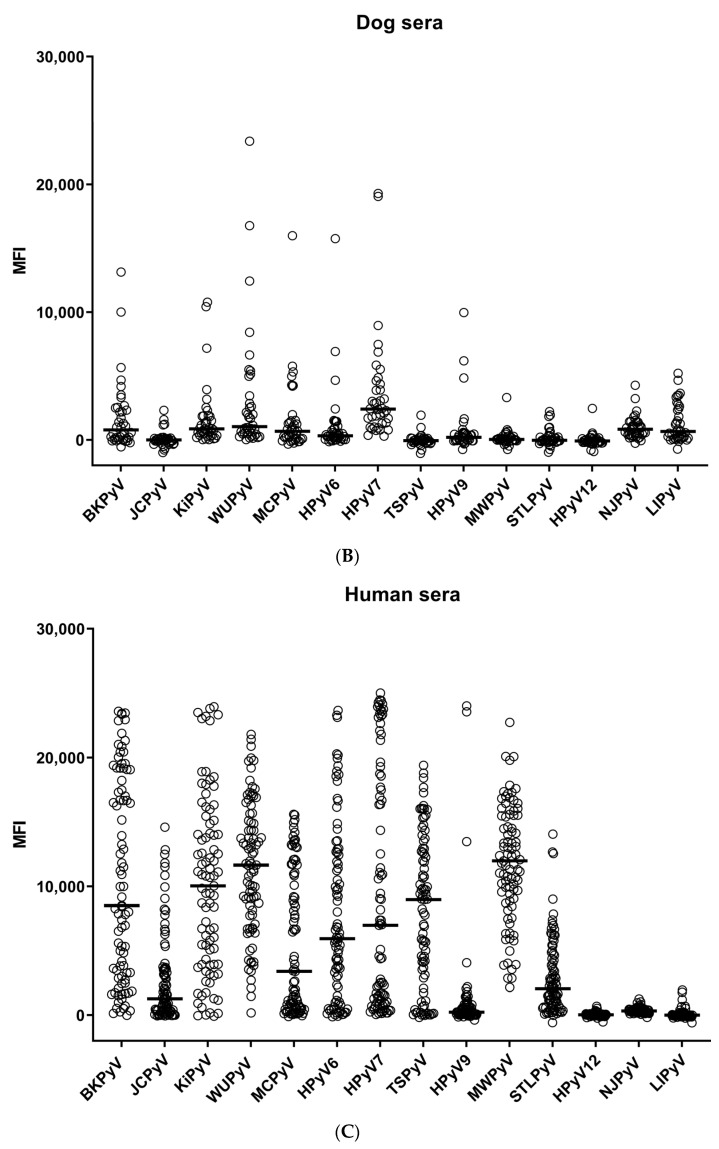
(**A**–**C**) IgG seroresponses against VP1 of 14 HPyVs measured in cat (**A**), dog (**B**), and human (**C**) sera. The horizontal lines indicate the median fluorescence intensity measured against each virus.

A total of 40 cat serum samples, 40 dog serum samples, and 87 human serum samples were tested in the immunoassay. Two dog serum samples were excluded from the analysis due to high background reactivity. The HPyV seroresponses measured in cats were generally low, except for those directed against LIPyV (Figure 1A). Seroresponses in dogs were low against all HPyVs (Figure 1B), with some occasional seroreactivity seen against different HPyVs. The human HPyV seroresponses were high, except for HPyV12, NJPyV and LIPyV (Figure 1C).

**Figure 2:**
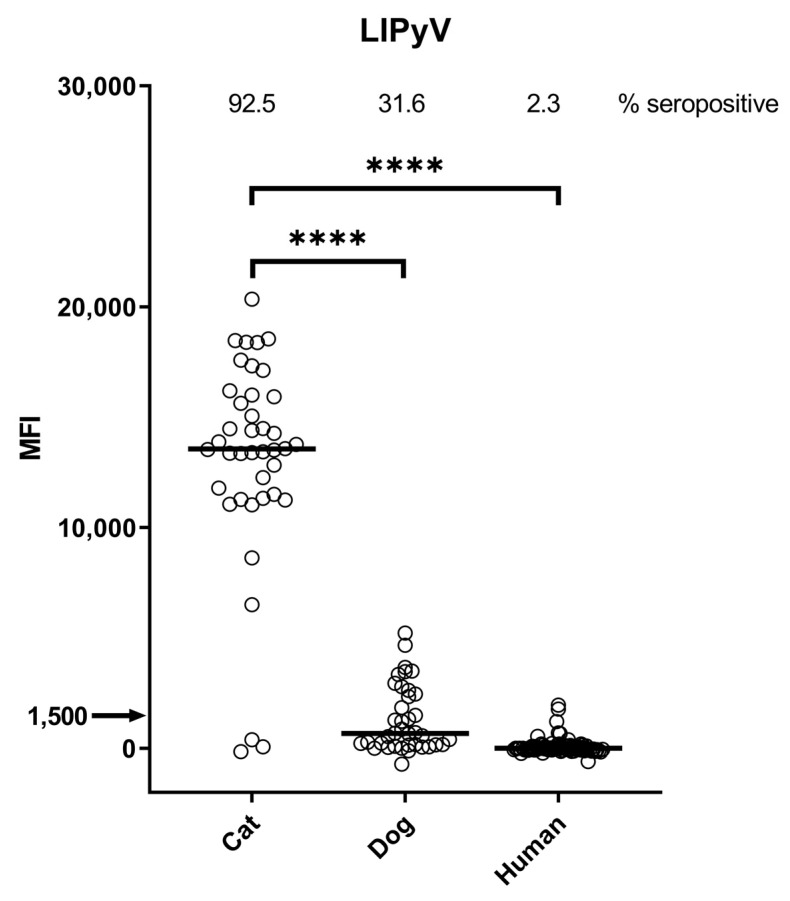
IgG seroresponses against VP1 of LIPyV measured in cat, dog, and human sera. The horizontal lines indicate the median fluorescence intensity. The arrow indicates the arbitrary cut-off value above which a serum is considered seropositive. The percentage seropositive is shown in the top of the graph. Significance level **** indicates *p*-value < 0.0001 in Mann–Whitney U test.

Antibody titres for LIPyV were much higher in cats (13,552 MFI; 95% CI: 12821–14486) when compared to dogs (666 MFI; 95% CI: 220–1484) and humans (−6 MFI; 95% CI: −27–10) (Mann–Whitney U test, both *p* < 0.0001) (Figure 2). By applying an arbitrary cut-off of MFI 1500, the LIPyV seroprevalence was calculated as 92.5% for cats, 31.6% for dogs, and 2.3% for humans.

**Figure 3:**
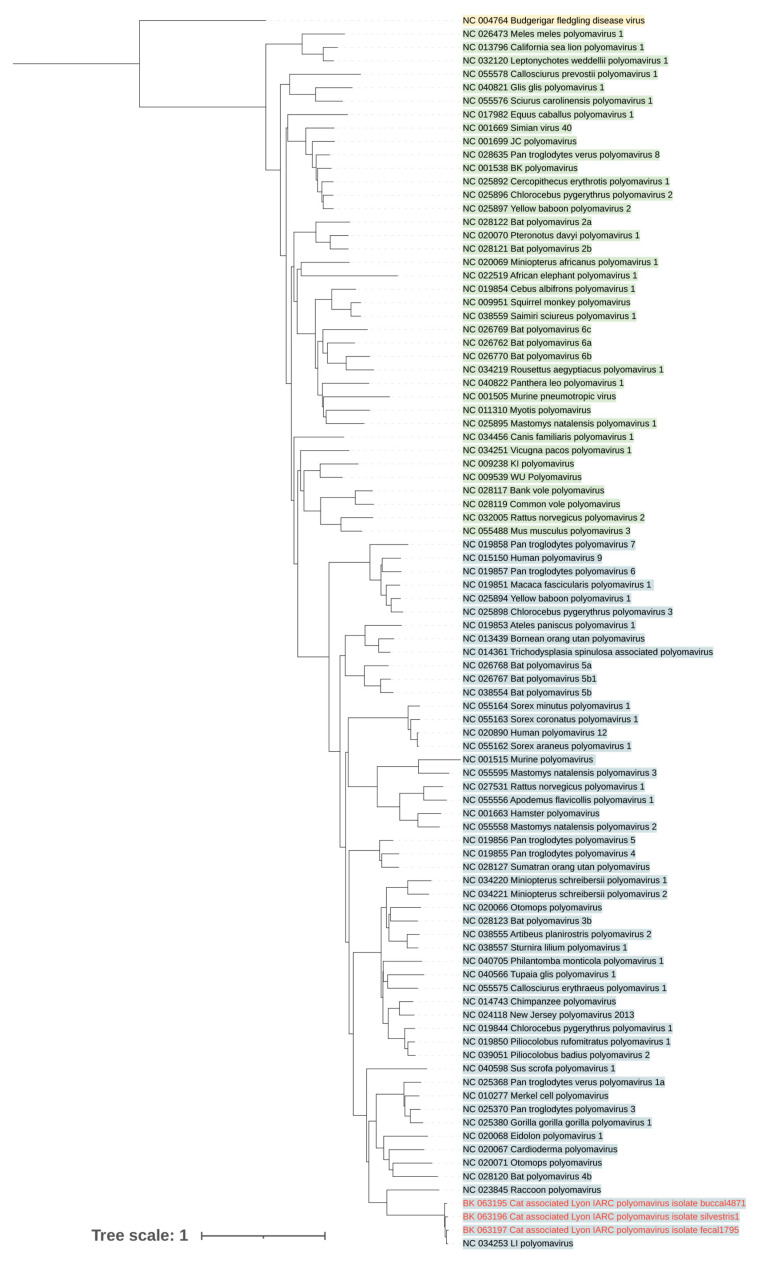
Phylogenetic tree based on nucleic acid sequences of the LT coding region of *Alphapolyomaviruses* (blue), *Betapolyomaviruses* (green), and a single *Gammapolyomavirus*, which is used as an outgroup to root the tree. The three new polyomavirus sequences (text in red) strongly cluster together with the original Lyon IARC polyomavirus sequence (NC_034253). Branch lengths represent the expected number of substitutions per site.

To further strengthen the association between feline hosts and LIPyV, the Sequence Read Archive database was searched for novel LIPyV-related genomes. Three genomes were discovered in samples originating from *Felis catus* (BK063195), *Felis silvestris* (BK063196), and a cat faecal sample (BK063197). A phylogenetic tree for select polyomavirus Large T antigen (LT) protein sequences is shown in Figure 3. The new feline-associated sequences cluster with the original LIPyV sequence.

## 4. Discussion

By comparing the seroprevalence and intensity of IgG seroresponses against all known HPyVs between cats, dogs, and humans, we provide strong evidence that LIPyV is not a human-tropic polyomavirus. High LIPyV seroresponses were observed only in cats. Together with the described detection of LIPyV variants in domestic and wild cat species [22,23] and the virtual lack of LIPyV DNA detection in samples from humans and other primates, all available evidence suggests that felines are the most likely natural host of LIPyV. It appears unlikely that humans are common zoonotic hosts for the virus. It seems likely that the original detection reflected environmental contamination with cat-derived virions.

Detection of virus-specific antibody responses, which usually arise only after genuine infection, could prevent such false interpretations and contribute to correct natural host calling and species classification. By using an arbitrary cut-off, we have determined a seroprevalence of 92.5% in cats, which is much higher than for dogs and humans. Although 31.6% of dog serum samples were also seropositive, both the median MFI value (666 MFI) and the highest observed MFI value (5211) were remarkably lower than those observed in the cats (13,552 and 20,348, respectively). The lower-magnitude seroresponsiveness of dogs could reflect the existence of an as yet undetected dog-specific LIPyV-like virus. In humans, barely any seroreactivity against LIPyV was detected, comparable to what we published previously [18].

Although an older serological study [33] suggested that some humans might be productively infected with bovine polyomavirus 1 and more recent reports have documented polyomavirus transmission between closely related bat species [34], a general model [25] is that polyomaviruses tend to co-evolve with their mammalian hosts and cross-species transmission of polyomavirus infections is rare. One example of the concept is SV40, a rhesus macaque polyomavirus that inadvertently contaminated early lots of poliovirus vaccines. Despite the fact that millions of individuals were exposed to SV40, there is no clear evidence conclusively documenting transmission of the virus among humans [35].

Whether primary feline LIPyV infection is accompanied by diarrhoea or other disease should be the subject of further investigation. A near relative of LIPyV, raccoon polyomavirus 1, is thought to be the cause of an apparent outbreak of brain tumours in the western United States [36]. It would thus be interesting to search for LIPyV sequences in cat tumour samples.

## Figures and Tables

**Table 1:** Human polyomaviruses analysed in this study (adapted from [2]).

Name (Acronym)	Species	Disease or Symptom Associated with Infection	Seroprevalence, % ^1^	Year and Specimen of Virus Discovery (Reference)
BK polyomavirus (BKPyV)	*Betapolyomavirus hominis*	Transplant nephropathy; Hemorrhagic cystitis	99	1971, Urine [3]
JC polyomavirus (JCPyV)	*Betapolyomavirus secuhominis*	Progressive multifocal leukoencephalopathy (PML)	62	1971, Brain [4]
Karolinska Institutet polyomavirus (KIPyV)	*Betapolyomavirus tertihominis*	Respiratory illness	92	2007, Nasopharynx [5]
Washington University polyomavirus (WUPyV)	*Betapolyomavirus quartihominis*	Respiratory illness	99	2007, Nasopharynx [6]
Merkel cell polyomavirus (MCPyV)	*Alphapolyomavirus quintihominis*	Merkel cell carcinoma	82	2008, Skin [7]
Human polyomavirus 6 (HPyV6)	*Deltapolyomavirus sextihominis*	Pruritic and dyskeratotic dermatosis	83	2010, Skin [8]
Human polyomavirus 7 (HPyV7)	*Deltapolyomavirus septihominis*	Pruritic and dyskeratotic dermatosis	71	2010, Skin [8]
Trichodysplasia spinulosa polyomavirus (TSPyV)	*Alphapolyomavirus octihominis*	Trichodysplasia spinulosa	79	2010, Skin [9]
Human polyomavirus 9 (HPyV9)	*Alphapolyomavirus nonihominis*	None	19	2011, Serum [10,11]
Malawi polyomavirus (MWPyV)	*Deltapolyomavirus decihominis*	None	100	2012, Feces [12,13]
Saint Louis polyomavirus (STLPyV)	*Deltapolyomavirus undecihominis*	None	65	2012, Feces [14]
Human polyomavirus 12 (HPyV12)	*Sorex araneus polyomavirus 1*	None	4	2013, Liver [15]
New Jersey polyomavirus (NJPyV)	*Alphapolyomavirus terdecihominis*	Vasculitis, myositis, retinitis	5	2014, Muscle [16]
Lyon IARC polyomavirus (LIPyV)	*Alphapolyomavirus quardecihominis*	None	6	2017, Skin [17]

^1^ As determined and described previously [18].

## Data Availability

Data available upon request.
